# The clinical value of ultrasound in assessing ovarian strangulation in female infants and toddlers with ovarian hernia

**DOI:** 10.3389/fped.2024.1366516

**Published:** 2024-05-22

**Authors:** Jiaojiao Gu, Chen Liu

**Affiliations:** Department of Medical Ultrasonics, Guangzhou Women and Children's Medical Center, Guangzhou Medical University, Guangzhou, China

**Keywords:** ovarian hernia, ultrasound, infants and toddlers, ovarian strangulation, emergency

## Abstract

**Objective:**

To explore the clinical value of ultrasound examination in evaluating the presence of ovarian torsion in female infants with inguinal ovarian hernia.

**Methods:**

We conducted a retrospective analysis of 91 cases of ovarian hernia diagnosed by ultrasonography at our institution. Among them, 6 cases were identified as ovarian strangulation, while 85 cases were classified as non-ovarian strangulation. All cases underwent high-frequency ultrasound examination. We analyzed whether there were differences in the associated factors between the two groups and compared the disparities in the urgency of surgery between the two groups.

**Results:**

Significant differences were observed between the ovarian strangulation group and the non- strangulation group in terms of ovarian volume, ovarian blood flow, and the presence of incarceration. The need for emergency surgery was significantly associated with the presence of incarceration and ovarian torsion.

**Conclusion:**

Ultrasound has good clinical value in diagnosing ovarian hernia and determining the presence of ovarian strangulation. It can assist clinical physicians in determining the timing of surgery for children with ovarian hernia.

## 1 Introduction

Inguinal hernia, particularly inguinal hernia with ovarian involvement, is a common condition in pediatric patients, with an incidence ranging from 0.8% to 4%, and reaching approximately 30% in premature infants (1). The contents of an inguinal hernia may include the small intestine, bladder, omentum, testis, ovary, fallopian tube, and uterus. Although inguinal hernias are more prevalent in male children, they are not uncommon in female infants. Unlike hernias in males, which often involve the omentum and intestines, hernias in female patients may contain organs such as the ovary, fallopian tube, and uterus, with the incidence of ovarian hernia ranging from 15% to 30%. Ovarian hernias pose a risk of ovarian torsion, a condition frequently caused by ovarian twisting, with reported variations in its incidence (2). Failure to promptly address torsion may result in irreversible consequences for the child's future reproductive function.

High-frequency ultrasound is a commonly used imaging method for diagnosing ovarian hernias. Some scholars argue that sonographic diagnosis is considered to be almost 100% effective in cases of inguinal hernia (3, 4). While some studies suggest that CT imaging can achieve diagnostic efficacy comparable to ultrasound in ovarian hernia diagnosis (5), ultrasound is often preferred as the initial imaging method in pediatric patients due to its simplicity, shorter processing time, and lack of radiation (6).

Currently, the treatment of inguinal hernias primarily involves manual reduction and surgical intervention. Esposito et al. (7) propose that laparoscopic hernia repair should be considered the optimal approach for treating inguinal hernias with ovarian involvement in female infants. Clinical decision-making regarding the choice of reduction method and the urgency of surgical intervention is primarily based on assessing clinical signs of hernia incarceration and torsion. The primary objective of this study is to explore ultrasound features associated with ovarian torsion in ovarian hernias, aiming to assist clinicians in determining the urgency of the condition in pediatric patients, aiding in the formulation of diagnostic and treatment plans, and assessing the need for emergency surgical intervention.

## 2 Materials and methods

A retrospective analysis was conducted on female pediatric patients diagnosed with inguinal hernia, including ovarian involvement, through ultrasound examinations at our department from January 2020 to December 2022. A total of 89 cases were included and two cases presented with bilateral inguinal hernias containing ovaries, resulting in a total of 91 cases of ovarian hernia. Inclusion criteria were as follows: (1) female infants and toddlers (age < 3 years); (2) underwent high-frequency ultrasound examinations in our department, confirming hernia contents including ovaries; (3) received surgical treatment during the follow-up period. Exclusion criteria included: (1) indeterminate gender or genetic/chromosomal abnormalities, such as androgen insensitivity syndrome and Mayer-Rokitansky-Kuster-Hauser syndrome (8); (2) hernias treated solely by manual reduction; (3) elective surgeries where hernia contents did not include ovaries; (4) incomplete clinical data. This study has obtained ethical approval from the institutional review board.

Two ultrasound physicians with over five years of experience retrospectively analyzed ultrasound images within our Picture Archiving and Communication System (PACS) that met the inclusion criteria. Images were obtained using high-frequency linear array probes from Canon Aplio500, Philips EPIQ7, GE Logiq 11, and Mindray Resona8 color Doppler ultrasound diagnostic equipment. Image selection criteria included: (1) clear visualization of the two-dimensional grayscale and color Doppler images of the largest longitudinal section of the ovary in the inguinal region, measuring the longitudinal and transverse diameters; (2) clear display of the transverse two-dimensional grayscale image of the ovary in the inguinal region, measuring the width diameter; (3) clear visualization of the internal ring of the inguinal canal, measuring the inner ring diameter.

Ovarian volume was estimated using the ellipsoid formula: Ovarian volume (cm^3^) = 0.52 * Length (cm) * Width (cm) * Thickness (cm); the longitudinal-to-transverse ratio was defined as the length diameter/thickness. All patients underwent laparoscopic inguinal hernia repair, and ovarian torsion was determined based on intraoperative examination, confirming the presence or absence of ovarian torsion.

Statistical analysis was performed using SPSS 24.0. Descriptive statistics are presented as $\bar{x}\pm s$ for continuous variables, and *t*-tests were applied. Frequency counts were used for categorical variables, analyzed using *χ*^2^ tests. If any cell had an expected count of less than 5, a continuity correction was applied. Binary logistic regression analysis was employed for multifactorial analysis.

## 3 Results

**Figure 1:**
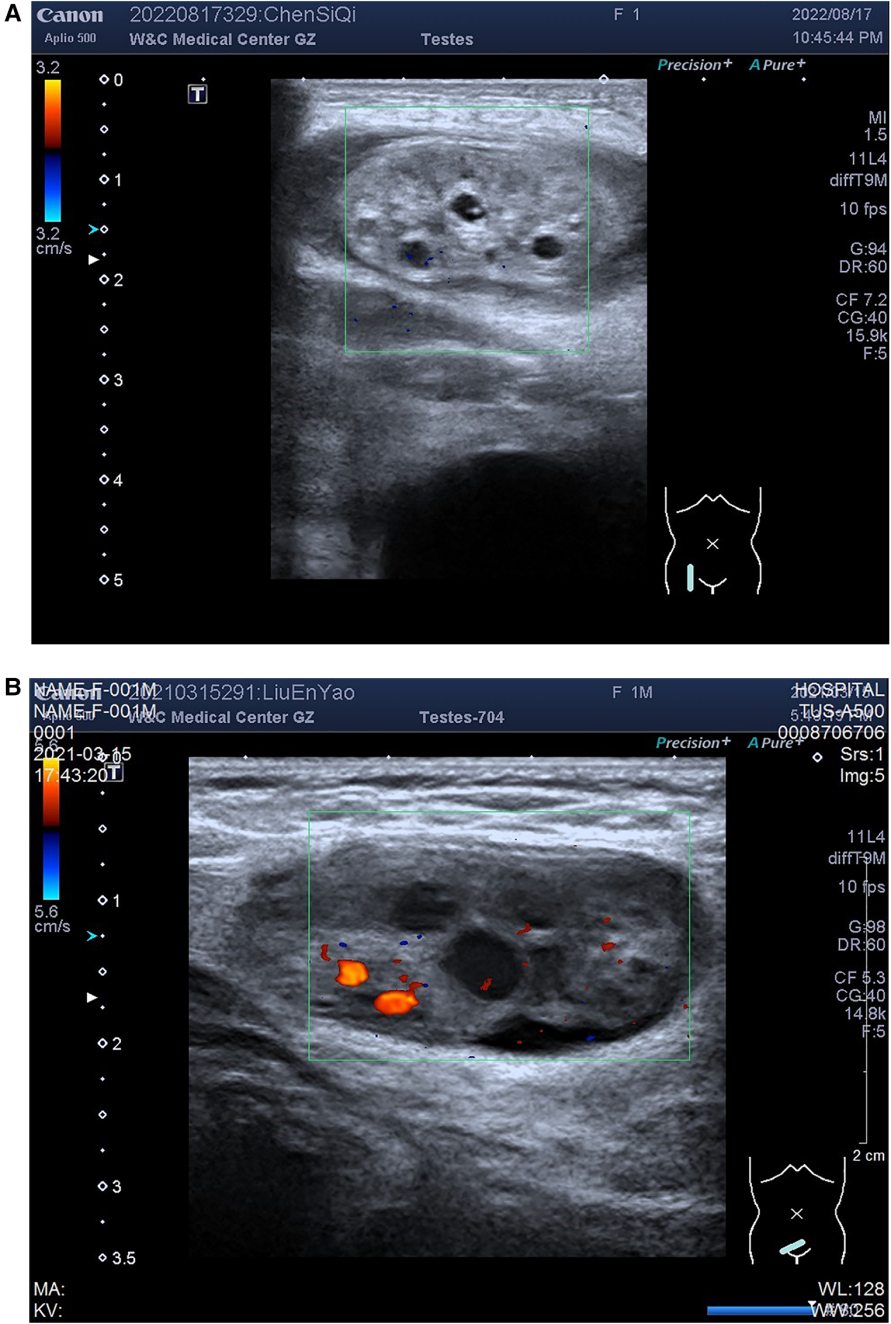
Ovarian images within hernias: (**A**) ovarian torsion; (**B**) Non-torsion. Figure illustrates ultrasound images of ovaries within hernias, showing (**A**) ovarian plump with uneven enhancement of parenchymal echogenicity, and Color Doppler Flow Imaging (CDFI) does not demonstrate obvious distribution of blood flow signals; (**B**) non-torsion case: ovarian morphology appears normal with homogeneous parenchymal echogenicity, and CDFI reveals a distributed blood flow signal.

The age of the 89 pediatric patients with ovarian hernia ranged from 17 days to 3 years. Among them, 68 were infants (age ≤1 year, 76.4%), and 21 were toddlers (age >1 year and age ≤3 years, 23.6%), with two infants having bilateral ovarian hernias. 48 cases were clinically diagnosed as incarcerated hernias, and 43 cases were deemed reducible hernias. Six cases exhibited enlarged and congested ovaries with torsion during surgery, classified as the torsion group, while the remaining 85 cases had normal ovaries without significant ischemic changes, and were categorized into the non-torsion group. Typical ultrasound images of ovarian torsion and non-torsion in ovarian hernias are shown in Figure 1. A comparison of clinical data and ultrasound features between the ovarian torsion and non-torsion groups is presented in Table 1.

**Table 1:** Clinical characteristics and ultrasound features comparison between the torsion and non-torsion groups.

		Torsion group	Non-torsion group	*P* value
Ovarian volume (cm^3^)		5.17 ± 1.76	1.66 ± 0.75	<0.001
Ovarian L/W ratio		1.80 ± 0.53	2.14 ± 0.50	0.490
Internal ring diameter (mm)		7.58 ± 1.91	6.78 ± 2.40	0.671
Ovarian blood flow	Present	2	74	0.004*
	Absent	4	11	
Incarceration hernia	Yes	6	42	0.048*
	No	0	43	
Hernia sac location	Right	2	32	1.000*
	Left	4	53	
Age group	Infant	4	66	0.908*
	Toddler	2	19	

* Continuous correction was applied when there was an expected count less than 5 in any cell.

Significant differences were observed between the ovarian torsion and non-torsion groups in ovarian volume (*P* < 0.001), ovarian blood flow (*P* = 0.04), and the presence of incarcerated hernias (*P* = 0.048). However, no significant statistical differences were found in ovarian longitudinal-to-transverse ratio (*P* = 0.490), inguinal internal ring diameter (*P* = 0.671), patient age (*P* = 0.908), and hernia sac location (*P* = 1.000). Using the presence of ovarian torsion as the dependent variable (torsion = 1, non-torsion = 0), factors with significant differences in the above univariate analysis, including ovarian volume, ovarian blood flow, and the presence of incarcerated hernias, were used as independent variables to establish a binary logistic regression equation, with only ovarian volume included in the equation, as shown in Table 2.

**Table 2:** Results of binary logistic regression for factors associated with ovarian torsion.

Predictor	Coefficient	Standard error	Wald value	*P*-value	Odds ratio
Ovarian volume	2.79	1.06	6.93	0.008	16.33
Constant	−10.99	3.73	8.67	0.003	-

The relationship between patient age, the presence of ovarian hernia incarceration and torsion, and the clinical judgment of surgical urgency by physicians is detailed in Table 3. The decision of whether to perform emergency surgery at our institution is significantly correlated with the presence of hernia incarceration and torsion, while no clear statistical correlation was found with patient age. All cases in the ovarian torsion group were confirmed to have ovarian torsion during surgery, with a torsion angle ranging from 180 degrees to 720 degrees. Ovarian detorsion was performed in all cases, preserving the ovaries without any cases of ovarian removal. All cases underwent at least one pelvic ultrasound follow-up during the observation period, showing satisfactory ovarian blood flow in all instances.

**Table 3:** Relationship between patient age, ovarian herniation, torsion, and the urgency of surgery.

		Elective surgery	Emergency surgery	*χ*^2^ value	*P*-value
Incarceration hernia	Yes	11	37	42.093	<0.001
	No	39	4		
Ovarian torsion	Yes	0	6	5.638	0.018*
	No	50	35		
Age group	Infant	35	35	2.996	0.083
	Toddler	15	6		

* Continuous correction was applied when there was an expected count less than 5 in any cell.

## 4 Discussion

In the pediatric population, inguinal hernias occur due to the incomplete closure of the processus vaginalis during gonadal descent. The embryological mechanisms and triggers of ovarian hernias in female children lack a unified consensus. Traditionally, the mechanism of ovarian hernias in female children was thought to be similar to the descent of the testes in male children. Before the fifth week of fetal development, the gubernaculum (in males) or round ligament (in females) attaches to the tail of the gonadal complex from the base of the abdominal membrane on both sides. In males, the inguinal canal migrates towards the inguinal internal ring and the inner surface of the scrotum through the gubernaculum, completing this process by the 32nd week of fetal development. In females, this process occurs earlier, forming the Nuck canal by the eighth month of gestation. Failure of Nuck canal closure can lead to inguinal hernias. However, recent studies suggest that the mechanism of ovarian hernias in female children may differ from the descent of testes in male children (9–12). Hutson et al. (13) proposed that ovarian hernias form as a result of the ovarian ligament pulling the ovary into the sac extending from the parietal peritoneum to the inguinal region. Kuyama et al. (14) found that the round ligament in some infants with ovarian hernias was shorter and gradually lengthened with growth. They speculated that the occurrence of ovarian hernias might be associated with a shorter round ligament. Scheier et al. (15) suggested that premature birth (before Nuck canal closure), excessive length of the fallopian tube leading to ovarian mobility, and increased intra-abdominal pressure (including deep breathing or straining during bowel movements) could increase the risk of ovarian hernias in infants.

In our study, the number of ovarian hernias in the infant group (76.4%) exceeded that of the toddler group (23.6%), likely because their ovaries are located near the pelvic rim and the inguinal region, making ovarian hernias more likely. Lee et al. (16) also found in their study of 1,210 female children that the risk of ovarian hernia incarceration increases with younger age. In our study, all six cases of ovarian torsion were discovered intraoperatively, aligning with Chen et al.'s (17) research, which indicated that ovarian torsion is the most important cause but not the only cause of ovarian torsion.

In our study, among 91 cases of ovarian inguinal hernia, six cases experienced ovarian strangulation. The research conducted by Dreuning and colleagues revealed that among over a thousand cases of pediatric inguinal hernia, ovarian inguinal hernia was diagnosed in 21.7% of the patients, with 6% experiencing strangulation of the ovary (18). In our study, sonographic findings of ovarian torsion manifest as an increase in ovarian volume, accompanied by heterogeneous enhancement in echogenicity. Additionally, color Doppler imaging typically reveals an absence or sparsity of blood flow signals. The occurrence of ovarian torsion in pediatric patients with ovarian hernias is closely related to ovarian volume, ovarian blood flow, and the presence of hernia incarceration. Similar conclusions were drawn by Chen et al. (17), who found that among potential risk factors for ovarian torsion in pediatric patients with ovarian hernias, the association between ovarian volume and ovarian torsion was the strongest. Ovaries with a volume greater than 5 cm^3^ were particularly prone to torsion. Hernia incarceration refers to the compression of intra-abdominal organs in inguinal hernias. After ovarian entrapment, the volume of the ovary is enlarged, exhibiting resistance to reduction. Additionally (19), due to the ovarian volume being greater than the internal ring, it can lead to severe compression of the ovarian vascular pedicle, making ovarian torsion more likely. Boley et al. (20) elucidated that the ovarian pedicle undergoes constriction and elongation during incarceration, thereby heightening the susceptibility to ovarian torsion. Zeng et al. (21) argue that incarcerated ovaries have the potential to undergo torsion, thereby elevating the risk of strangulation. Merriman et al.'s (22) study found that incarcerated ovarian herniation increases the risk of torsion, resulting in the obstruction of normal venous and lymphatic return within the canal of Nuck. As the herniated sac exerts sustained vascular compromise, the ovary can undergo infarction with accompanying tissue necrosis. Moreover, the risk of ovarian torsion in incarcerated hernias is not only associated with compression. The risk of torsion caused by ovarian entrapment is significantly higher than that caused by compression alone. Furthermore, the risk of ovarian torsion increases with the angle of ovarian torsion (5). Although an enlarged ovary with no apparent blood flow is a crucial sign of ovarian torsion, ultrasound's sensitivity in accurately diagnosing ovarian torsion is limited. The small vascular volume, low blood flow, and deep location of the ovaries contribute to the limited accuracy of ultrasound assessment of ovarian blood flow. Furthermore, it is noteworthy that the ovary receives a dual blood supply from both the ovarian artery and the uterine artery. In many cases (23), arterial flow tends to persist for a considerable duration even after the loss of venous and lymphatic drainage. Chen et al.'s (11) study found that 44.4% of pediatric patients with ovarian torsion had undetectable blood flow signals on ultrasound, emphasizing the challenge in accurately assessing ovarian blood flow in torsion cases.

The assessment of the risk of torsion and the timing of surgery in pediatric patients with ovarian hernias remain inconclusive. Some argue that the diagnosis should be made before ovarian tissue necrosis, protecting infants and toddlers from catastrophic consequences. The time interval between ovarian hernia incarceration and subsequent torsion remains unclear. Ovaries within an incarcerated hernia are at a higher risk of torsion. Merriman et al. (22) recommended immediate surgical repair within 24–48 h for all pediatric patients with ovarian hernias to prevent torsion and necrosis. In their study, the rate of ovarian torsion was 19%. In the study by Lee et al. (24), oophorectomy was successfully avoided in all but one patient. This positive outcome was attributed to the fact that all surgeries were conducted within 24 h of diagnosing an incarcerated hernia. The rate of ovarian torsion in this study is higher compared to previous research, standing at 28.9%. However, other researchers hold different views. Hirabayashi et al. (2) suggested that ovarian hernias in newborns and infants might spontaneously reduce to the abdomen in late infancy, even for those with irreducible hernias, elective surgery could be considered after nine months with parental consent. In their study, no cases of ovarian torsion were found. Fukuhara et al. (25) observed that 22 out of 37 cases of ovarian hernias self-reduced, with a median age of spontaneous regression at 242–262 days. They proposed that, due to the high incidence of ovarian hernias in infancy, an observation strategy until six months might be considered, and if not resolved, elective surgery could be performed. There have been reports indicating that performing surgery in early infancy may lead to anesthetic and perioperative complications, including occurrences such as apnea, bradycardia, and hernia recurrence (26). The existing studies have not adequately differentiated the timing of ovarian torsion occurrence. However, our research enables the assessment of ovarian torsion during examinations based on ultrasound features. This capability assists clinical practitioners in making optimal decisions in patient care. Early diagnosis of ovarian torsion therefore is crucial to be able to protect the gonadal tissue and maintain fertility.

In our study, all ovaries were preserved, including the six cases of ovarian torsion, consistent with the findings of Hughes et al. (27). They advocated for preserving ovaries in all cases, regardless of the surgeon's assessment of ischemia during surgery. They observed functional ovarian tissue in all patients during follow-up. Current literature and guidelines recommend detorsion and preservation of ovaries after torsion, rather than ovarian removal. There is no clear evidence that thromboembolism occurs after ovarian torsion (28).

Additionally, there have been reported cases of pediatric patients with ovarian hernias, where postoperative procedures ensured the complete preservation of the ovaries (15, 29). However, in some studies, some children with ovarian torsion underwent oophorectomy. Lee et al. (24) reported eight cases of ovarian torsion in pediatric patients with ovarian hernias, of which seven cases retained the ovaries, and one child underwent oophorectomy. Houben et al. (30) found three cases of ovarian torsion in pediatric patients with ovarian hernias, one of whom underwent oophorectomy. Dreuning et al. (18) reported 235 cases of girls with ovarian hernias in a study of 1,084 cases of groin hernias. Fourteen cases experienced ovarian torsion during the perioperative period, and one case required salpingo-oophorectomy due to complete ovarian necrosis. In a recent study, Wang et al. (31) described a case of a pediatric patient with ovarian hernia where manual reduction failed, leading to necrosis of the right ovary and fallopian tube, ultimately requiring oophorectomy. Kwang Ho Choi et al. also reported a case of ovarian hernia with ovarian torsion, necessitating the removal of the affected ovary (19).

This study has some limitations. Firstly, it is a retrospective study, and some clinical information was incomplete in certain cases, leading to the exclusion of potentially relevant clinical information such as the number of organ herniations, the number of manual reduction attempts, and the time interval between diagnosis and surgery.

## 5 Conclusion

In conclusion, ovarian hernias in infant and toddler females are not uncommon. Ultrasound plays a valuable role in the diagnosis of ovarian hernias and the assessment of ovarian torsion. Ovarian volume enlargement, disappearance of ovarian blood flow, and ovarian incarceration are associated with ovarian torsion, with ovarian volume enlargement being the most strongly correlated. Ultrasound examinations can assist clinicians in determining the timing of surgery for pediatric patients with ovarian hernias.

## Data Availability

The original contributions presented in the study are included in the article/Supplementary Material, further inquiries can be directed to the corresponding author.

## References

[1] Esposito C Escolino M Turrà F Roberti A Cerulo M Farina A Current concepts in the management of inguinal hernia and hydrocele in pediatric patients in laparoscopic era. Semin Pediatr Surg. (2016) 25(4):232–40. 10.1053/j.sempedsurg.2016.05.006 27521714

[2] Hirabayashi T Ueno S Hirakawa H Tei E Mori M. Surgical treatment of inguinal hernia with prolapsed ovary in young girls: emergency surgery or elective surgery. Tokai J Exp Clin Med. (2017) 42(2):89–95. Published July 20, 2017. 28681369

[3] Yang DM Kim HC Lim JW Jin W Ryu CW Kim GY Sonographic findings of groin masses. J Ultrasound Med. (2007) 26(5):605–14. 10.7863/jum.2007.26.5.605 17460003

[4] Jedrzejewski G Stankiewicz A Wieczorek AP. Uterus and ovary hernia of the canal of nuck. Pediatr Radiol. (2008) 38(11):1257–8. 10.1007/s00247-008-0959-x 18688608

[5] Hyun PM Jung AY Lee Y Yang I Yang DH Hwang JY. CT and US findings of ovarian torsion within an incarcerated inguinal hernia. Emerg Radiol. (2015) 22(1):91–4. 10.1007/s10140-014-1248-7 24917391

[6] Kitami M. Ultrasonography of pediatric urogenital emergencies: review of classic and new techniques. Ultrasonography. (2017) 36(3):222–38. 10.14366/usg.17011 28494525 PMC5494863

[7] Esposito C Gargiulo F Farina A Del Conte F Cortese G Servillo G Laparoscopic treatment of inguinal ovarian hernia in female infants and children: standardizing the technique. J Laparoendosc Adv Surg Tech A. (2019) 29(4):568–72. 10.1089/lap.2018.0630 30676267

[8] Saini R Bains L Kaur T Lal P Pal V Beg MY Ovarian inguinal hernia—a possibility in MURCS syndrome. J Ovarian Res. (2021) 14(1):114. Published September 3, 2021. 10.1186/s13048-021-00869-y 34474687 PMC8414687

[9] George EK Oudesluys-Murphy AM Madern GC Cleyndert P Blomjous JG. Inguinal hernias containing the uterus, fallopian tube, and ovary in premature female infants. J Pediatr. (2000) 136(5):696–8. 10.1067/mpd.2000.105140 10802507

[10] Yigit H Tuncbilek I Fitoz S Yigit N Kosar U Karabulut B. Cyst of the canal of nuck with demonstration of the proximal canal: the role of the compression technique in sonographic diagnosis. J Ultrasound Med. (2006) 25(1):123–5. 10.7863/jum.2006.25.1.123 16371563

[11] Park SJ Lee HK Hong HS Kim HC Kim DH Park JS Hydrocele of the canal of nuck in a girl: ultrasound and MR appearance. Br J Radiol. (2004) 77(915):243–4. 10.1259/bjr/51474597 15020367

[12] Ming YC Luo CC Chao HC Chu SM. Inguinal hernia containing uterus and uterine adnexa in female infants: report of two cases. Pediatr Neonatol. (2011) 52(2):103–5. 10.1016/j.pedneo.2011.02.006 21524631

[13] Hutson JM Kearsey I. Is the ovary in an inguinal hernia ‘descended’ like a testis or not? J Pediatr Surg. (2016) 51(7):1197–200. 10.1016/j.jpedsurg.2015.09.0141200 26463501

[14] Kuyama H Uemura S Yoshida A Yamamoto M. Close relationship between the short round ligament and the ovarian prolapsed inguinal hernia in female infants. Pediatr Surg Int. (2019) 35(5):625–9. 10.1007/s00383-019-04465-6 30863916

[15] Scheier E. Inguinal ovarian hernia on point of care ultrasound: case reports and review of the literature. Emerg Radiol. (2022) 29(1):215–7. 10.1007/s10140-021-01981-8 34427854

[16] Lee SR. Efficacy of laparoscopic herniorrhaphy for treating incarcerated pediatric inguinal hernia. Hernia. (2018) 22(4):671–9. 10.1007/s10029-017-1655-2 28852857

[17] Chen Y Peng XZ Lu W Zheng K Guo J Nie H Risk factors for strangulated ovarian hernia in female infants: the role of ovarian volume. Curr Med Sci. (2018) 38(6):1032–7. 10.1007/s11596-018-1980-4 30536066

[18] Dreuning KM Barendsen RW van Trotsenburg AP Twisk JW Sleeboom C van Heurn LE Inguinal hernia in girls: a retrospective analysis of over 1000 patients. J Pediatr Surg. (2020) 55(9):1908–13. 10.1016/j.jpedsurg.2020.03.015 32317102

[19] Choi KH Baek HJ. Incarcerated ovarian herniation of the canal of nuck in a female infant: ultrasonographic findings and review of literature. Ann Med Surg (Lond). (2016) 9:38–40. Published June 18, 2016. 10.1016/j.amsu.2016.06.003 27408712 PMC4925906

[20] Boley SJ Cahn D Lauer T Weinberg G Kleinhaus S. The irreducible ovary: a true emergency. J Pediatr Surg. (1991) 26(9):1035–8. 10.1016/0022-3468(91)90668-j 1941479

[21] Zeng K Murphy J Wilson EE. Female reproductive structures found in inguinal hernia sacs: a retrospective review. J Pediatr Surg. (2019) 54(10):2134–7. 10.1016/j.jpedsurg.2019.03.018 31036370

[22] Merriman TE Auldist AW. Ovarian torsion in inguinal hernias. Pediatr Surg Int. (2000) 16(5-6):383–5. 10.1007/s003830000428 10955568

[23] Oltmann SC Fischer A Barber R Huang R Hicks B Garcia N. Cannot exclude torsion–a 15-year review. J Pediatr Surg. (2009) 44(6):1212–7. 10.1016/j.jpedsurg.2009.02.028 19524743

[24] Lee SR. Ovarian incarceration and torsion in single-ovary versus multiple-reproductive organ prolapse in female inguinal hernia: a retrospective study of 510 infants who underwent laparoscopic hernia repair. J Laparoendosc Adv Surg Tech A. (2021) 31(1):110–6. 10.1089/lap.2020.0531 32931354

[25] Fukuhara M Onishi S Handa N Sato T Esumi G. Spontaneous reduction age for ovarian hernia in early infancy. Pediatr Int. (2022) 64(1):e15024. 10.1111/ped.15024 34643013

[26] Krishna G Emhardt JD. Anesthesia for the newborn and ex-preterm infant. Semin Pediatr Surg. (1992) 1(1):32–44. 1345468

[27] Hughes P Abdelhafeez A Byrne AT Real D Gillick J. A paediatric hernia with a twist: the presentation, imaging findings and management of a strangulated ovarian hernia. Ir Med J. (2015) 108(9):282–3. 26625655

[28] Dasgupta R Renaud E Goldin AB Baird R Cameron DB Arnold MA Ovarian torsion in pediatric and adolescent patients: a systematic review. J Pediatr Surg. (2018) 53(7):1387–91. 10.1016/j.jpedsurg.2017.10.053 29153467

[29] Aydin R Polat AV Ozaydin I Aydin G. Gray-scale and color Doppler ultrasound imaging findings of an ovarian inguinal hernia and torsion of the herniated ovary: a case report. Pediatr Emerg Care. (2013) 29(3):364–5. 10.1097/PEC.0b013e318285465c 23462392

[30] Houben CH Chan KW Mou JW Tam YH Lee KH. Irreducible inguinal hernia in children: how serious is it? J Pediatr Surg. (2015) 50(7):1174–6. 10.1016/j.jpedsurg.2014.10.018 25783312

[31] Wang YJ Chen L Zhang QL Zhang JQ Cui X Zhou CM. Single-site laparoscopic high ligation of the extraperitoneal hernia sac with an epidural needle for incarcerated ovarian hernia in infants. BMC Surg. (2022) 22(1):67. Published February 23, 2022. 10.1186/s12893-022-01520-3 35197030 PMC8867844

